# Association between cervical dysplasia and female genital schistosomiasis diagnosed by genital PCR in Zambian women

**DOI:** 10.1186/s12879-021-06380-5

**Published:** 2021-07-17

**Authors:** H. Rafferty, A. S. Sturt, C. R. Phiri, E. L. Webb, M. Mudenda, J. Mapani, P. L. A. M. Corstjens, G. J. van Dam, A. Schaap, H. Ayles, R. J. Hayes, L. van Lieshout, I. Hansingo, A. L. Bustinduy

**Affiliations:** 1 Department of Clinical Research, London School of Hygiene and Tropical Medicine, Keppel Street, London, WC1E 7HT UK; 2 Zambart, Lusaka, Zambia; 3 MRC International Statistics and Epidemiology Group, London School of Hygiene and Tropical Medicine, London, UK; 4 Livingstone Central Hospital, Livingstone, Zambia; 5 Department of Cell and Chemical Biology, Leiden University Medical Center, Leiden, The Netherlands; 6 Department of Parasitology, Leiden University Medical Center, Leiden, The Netherlands

**Keywords:** Female genital schistosomiasis, Cervical dysplasia, *Schistosoma haematobium*, PCR, Urogenital schistosomiasis

## Abstract

**Background:**

Female genital schistosomiasis (FGS) is a neglected tropical gynaecological disease that affects millions of women in sub-Saharan Africa (SSA). FGS is caused by *Schistosoma haematobium*, a parasitic carcinogen involved in the pathogenesis of squamous cell carcinoma of the bladder. Cervical cancer incidence and mortality are highest in SSA, where pre-cancerous cervical dysplasia is often detected on screening with visual inspection with acetic acid (VIA). There are no studies evaluating the association between VIA positivity and FGS diagnosed by genital PCR.

**Methods:**

Women were recruited from the Bilharzia and HIV (BILHIV) study in Zambia a community-based study comparing genital self-sampling to provider obtained cervicovaginal-lavage for the diagnosis of FGS in women aged 18–31. FGS was defined as positive *Schistosoma* DNA from any genital PCR. Urogenital schistosomiasis diagnostics included urine circulating anodic antigen, urine microscopy and portable colposcopy. Participants were offered cervical cancer screening using VIA at Livingstone Central Hospital. Associations of PCR confirmed FGS and other diagnostics with VIA positivity were assessed using multivariable logistic regression.

**Results:**

VIA results were available from 237 BILHIV participants. A positive *Schistosoma* PCR in any genital specimen was detected in 14 women (5.9%), 28.6% (4/14) of these women had positive VIA compared to 9.0% without PCR evidence of schistosome infection (20/223). *Schistosoma* PCR positivity in any genital specimen was strongly associated with VIA positivity (OR: 6.08, 95% CI: 1.58–23.37, *P* = 0.02).

**Conclusions:**

This is the first study to find an association between FGS and positive VIA, a relationship that may be causal. Further longitudinal studies are needed.

**Supplementary Information:**

The online version contains supplementary material available at 10.1186/s12879-021-06380-5.

## Introduction

Female genital schistosomiasis (FGS) is a neglected parasitic gynaecological disease that affects an estimated 56 million women in sub-Saharan Africa [1, 2]. It is most commonly caused by *Schistosoma haematobium*, blood flukes which lay eggs in the vesical plexus [2]. These eggs become entrapped in the urogenital mucosa resulting in inflammation and reproductive morbidity [3]. Cervical cancer is a leading cause of cancer-related deaths in African women [4–6]. In Zambia age-standardised incidence rates of cervical cancer are 63.3 per 100,000 with a mortality rate of 41.1 per 100,000, over 10 times that of the United States of America [4]. *S. haematobium* infection causes squamous cell carcinoma (SCC) of the bladder, therefore a role in cervical cancer pathogenesis is plausible [7]. This is a prescient prospect given the recent launch by WHO of a global initiative to eliminate cervical cancer by 2030 [8].

In the absence of a reference standard, making an FGS diagnosis can be challenging and often requires the use of multiple diagnostic modalities. FGS is associated with typical genital mucosal visible lesions, however parasite eggs can be found in macroscopically normal tissue [9, 10]. Sandy patches (grainy and homogenous) and abnormal blood vessels have been associated with *S. haematobium* eggs on pap smear [11]. Cervicovaginal lavage (CVL) or genital swabs can be used to detect *Schistosoma* DNA by PCR [12, 13]. Community-based approaches have shown that FGS diagnosis using self-collected cervical and vaginal swabs is acceptable to participants, has comparable sensitivity to CVL [12], and provides an objective alternative to imaging [14]. Urine microscopy for eggs or the detection of circulating anodic antigen (CAA) can diagnose active schistosome infection, but do not necessarily reflect genital involvement [12, 15].

Pre-cancerous stages of cervical cancer are treatable and can be detected with screening [16]. Human papillomavirus (HPV) testing is WHO-recommended for cervical screening as HPV infection is the most common cause of cervical cancer [17]. In resource-constrained settings where HPV testing is available, visual inspection with acetic acid (VIA) can be used to visualise pre-cancerous lesions [18]. In a large meta-analysis of 32 studies, including 18 in low and middle income countries, VIA was found to be less sensitive (0.69 vs. 0.95), but more specific (0.87 vs. 0.84) than HPV testing for the detection of cervical in situ neoplasia grade 2–3 as diagnosed by colposcopy with or without biopsy [19]. Identified lesions are treated with cryotherapy or loop electrosurgical excision procedure (LEEP). The co-existence of FGS and cervical malignancy is well-documented in pathology reports [20, 21], and homogenous sandy patches have been associated with the presence of high risk HPV [22]; however, this association is not universally reported [23]. This study aimed to evaluate the association between FGS and cervical dysplasia diagnosed by VIA in Zambian women.

## Materials and methods

This study was a community based cross-sectional study. Data previously collected for the bilharzia and HIV study (BILHIV) and stored in clinical records were analysed retrospectively.

### Participant recruitment

Between January and August 2018, 18–31 year-old, non-pregnant, sexually active participants in two schistosomiasis low-endemic communities in Zambia were consecutively recruited after the 36-month HPTN 071 (PopART) study visit to the bilharzia and HIV (BILHIV) study [12, 24].

Following informed consent, BILHIV study participants self-collected a urine sample, and cervical and vaginal swabs, as previously described [12]. During clinic follow-up at the cervical cancer clinic at Livingstone Central Hospital, midwives performed CVL and portable colposcopy (MobileODT, Tel Aviv, Israel) to capture images of vagina and cervix [12].

Cervicovaginal images were evaluated by one medically trained reviewer and classified as suggestive of ‘*visual FGS’* if homogenous sandy patches, grainy sandy patches, rubbery papules, or abnormal blood vessels were present, and negative if none were present [25]. Women with evidence of schistosome infection by any diagnostic, or by midwife’s clinical examination [25] were treated with 40 mg/kg praziquantel as recommended by WHO [25]. Participants with suspected sexually transmitted infections (STI) were offered syndromic management, as per Ministry of Health guidelines [26]. Routine STI testing was not performed at the point-of-care in this study. Laboratory-based fourth-generation HIV-1 testing (Abbott Architect HIV Ag/Ab Combo Assay, Wiesbaden, Germany) was performed for HPTN 071 (PopART) participants at each study visit [24].

### Visual inspection with acetic acid

Due to high community HIV prevalence [27], all women regardless of age, are routinely offered cervical cancer screening with VIA at the Cervical Cancer Clinic (CCC), in Livingstone Central Hospital by the Zambian Health Service. For those women that wished to be screened for cervical cancer and after CVL was obtained for the BILHIV study, 3–5% acetic acid was applied to the cervix. An opaque white reaction was classified as positive and no change was negative [18]. VIA results were documented in the clinical records at the CCC and were not collected as part of BILHIV. In line with national and local clinic protocols adapted to real-world resource limitations, HPV testing was not performed. Later, data matching was performed to identify BILHIV participants that had also attended the CCC for cervical cancer screening. All BILHIV participants with an available VIA result from routine Zambia Health Service screening were included if data could be matched from both sources.

### *Schistosoma* diagnostics

DNA isolation was performed at Leiden University Medical Center (LUMC), followed by real time-PCR for the detection of *Schistosoma* DNA, as previously described [12, 28, 29]. CAA quantification was performed using an up-converting reporter particle lateral flow assay (UCP-LF) at LUMC [30]. Analysing the equivalent of 417 μL urine, a CAA value of > 0.6 pg/mL was considered positive based on a series of negative controls (highest value plus 2 SDs) [30].

This study involved retrospective analysis of data collected for previously published work from the BILHIV study [12]. VIA data were collected from existing clinical records. No additional participant participation was required.

### Ethics

Ethical approval was granted by London School of Hygiene and Tropical Medicine (LSHTM) (reference 16,451) and University of Zambia Biomedical Research Ethics Committee (reference: 011–08-17). The Livingstone Central Hospital Superintendent gave permission to conduct the study.

### Statistical methods

Cervical dysplasia diagnosed by VIA was the dependent variable. Univariable logistic regression analysis was carried out to calculate crude odds ratios for associations between VIA and all exposure variables; multivariable logistic regression analysis was used to calculate adjusted odds ratios for associations between VIA and FGS variables controlling for age category and HIV status as a priori confounders. No further potential confounders could be included due to the small number of women with positive VIA. *P*-values were generated using likelihood ratio tests. All data were analysed using STATA 15 [31].

## Results

**Fig. 1:**
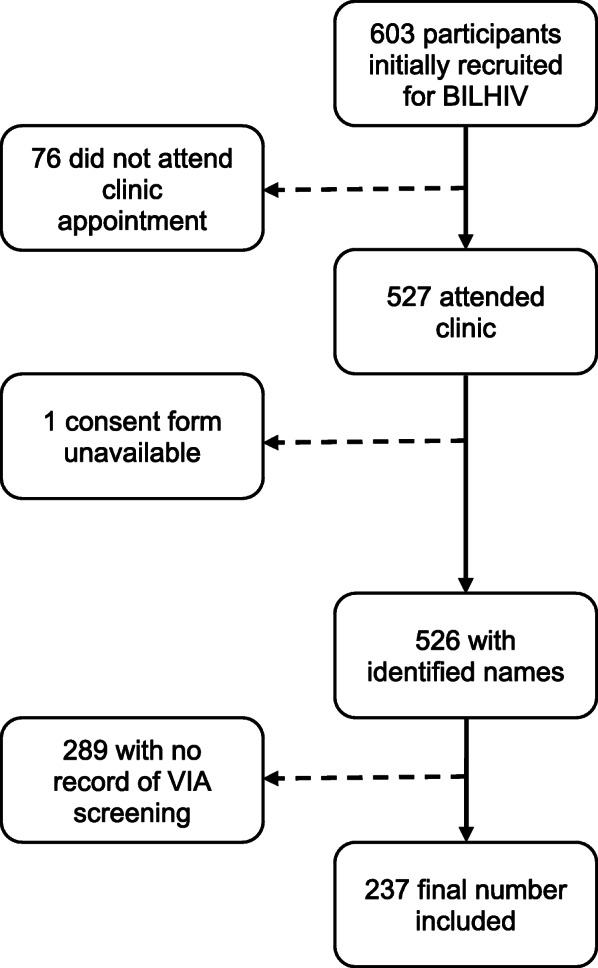
Participant recruitment of 237 women in the BILHIV study who underwent VIA

A total of 237/527 (44.9%) women from the BILHIV study also had VIA results available and were included in the analysis (see Fig. 1).

The median age was 24 (IQR 22–27) and the majority had some secondary education and were not currently employed. All 24 women (10.1%) with positive VIA (Table 1) were treated; 20 with cryotherapy (83.3%) and 4 with LEEP (16.7%). There was no association between any demographic factors and VIA positivity (Table 2).

**Table 1:** Baseline characteristics and diagnostic test results amongst 237 participant women

Characteristics		n (%)
Demography		
Age (years)	18–22	64 (27.0)
	23–26	91 (38.4)
	27–31	82 (34.6)
Education	None or primary	74 (31.2)
	Secondary or higher	163 (68.8)
Employment	Unemployed	163 (68.8)
	Employed	74 (31.2)
Marital status	Currently single	116 (49.0)
	Currently married	121 (51.1)
Ever pregnant	Never	36 (15.2)
	Yes	201 (84.8)
Contraception	Yes	183 (77.2)
	No	54 (22.8)
Previous bilharzia diagnosis or treatment	Yes	29 (12.2)
	Unsure	10 (4.2)
	No	198 (83.5)
HIV		
HIV status***	Positive	56 (23.8)
	Negative	179 (76.2)
HIV status self-reported****	Positive	42 (17.8)
	Negative	194 (82.2)
HIV seroconversion during HPTN 071 ***	Yes	5 (2.1)
	No	230 (97.9)
Cervical dysplasia		
VIA	Positive	24 (10.1)
	Negative	213 (89.9)
Treatment	No treatment	208 (87.8)
	Cryotherapy	20 (8.4)
	LEEP	4 (1.7)
	Antibiotics	5 (2.1)
*Schistosoma* diagnostics		
Genital *Schistosoma* PCR	Positive	14 (5.9)
	Negative	223 (94.1)
Imaging findings suggestive of FGS*	Present	70 (29.5)
	Absent	146 (61.6)
CAA**	Positive	35 (14.8)
	Negative	201 (84.8)
Urine microscopy	Positive	15 (6.3)
	Negative	222 (93.7)

*n = 216, 21 women with uninterpretable images; **n = 236, one urine vial arrived to LUMC empty; ***n = 235, 2 results missing from HPTN-271 (PopART) database; ****n = 236, one woman declined to disclose status

**Table 2:** Number (n) and percentage (%) of study variables amongst the study population by VIA status

		n (%)	n (%) VIA+	Crude OR VIA+	95% CI	*P*-value
FGS by PCR *n* = 237	Positive	14 (5.9)	4 (28.6)	4.06	1.15–14.38	**0.044**
	Negative	223 (94.1)	20 (9.0)	1		
Visual FGS *n* = 216^a^	Positive	70 (29.5)	5 (7.1)	0.63	0.22–1.79	0.364
	Negative	146 (61.6)	16 (11.0)	1		
CAA *n* = 236^b^	Positive	35 (14.8)	5 (14.3)		0.55–4.62	0.428
	Negative	201 (84.8)	19 (9.5)	1		
Urine microscopy *n* = 237	Positive	15 (6.3)	3 (20.0)	2.39	0.62–9.23	0.237
	Negative	222 (93.7)	21 (9.5)	1		
Age *n* = 237	18–22	64 (27.0)	5 (7.8)	1		0.112
	23–26	91 (38.4)	6 (6.6)	0.83	0.24–2.87	
	27–31	82 (34.6)	13 (15.9)	2.22	0.74–6.68	
HIV status *n* = 235^c^	Positive	56 (23.8)	8 (14.3)	1.7	0.68–4.23	0.265
	Negative	179 (76.2)	16 (8.9)	1		
District *n* = 237	Community A	142 (59.9)	15 (10.6)	1		0.785
	Community B	95 (40.1)	9 (9.5)	0.89	0.37–2.12	
Education *n* = 237	None or primary	74 (31.2)	11 (14.9)	1		0.113
	Secondary or higher	163 (68.8)	13 (8.0)	0.5	0.21–1.18	
Employment *n* = 237	Unemployed	163 (68.8)	15 (9.2)	1		0.490
	Employed	74 (31.2)	9 (12.2)	1.37	0.57–3.29	
Marital status *n* = 237	Currently single	116 (49.0)	14 (12.1)	1		0.331
	Currently married	121 (51.1)	10 (8.3)	0.66	0.28–1.55	
Ever pregnant *n* = 237	Never	36 (15.2)	20 (10.0)	0.88	0.28–2.76	0.834
	Yes	201 (84.8)	4 (11.1)	1		
Contraception *n* = 237	Yes	183 (77.2)	15 (8.2)	1		0.085
	No	54 (22.8)	9 (16.7)	2.24	0.91–5.50	
Condoms *n* = 237	Yes	47 (19.8)	6 (12.8)	1.4	0.52–3.75	0.514
	No	190 (80.2)	18 (9.5)	1		
Previous bilharzia diagnosis or treatment *n* = 237	Yes	29 (12.2)	3 (10.3)	1.09	0.30–3.94	0.630
	Unsure	10 (4.2)	2 (20.0)	2.36	0.46–12.00	
	No	198 (83.5)	19 (9.6)	1		

*Abbreviations*: *CAA* Circulating anodic antigen, *CI* Confidence interval, *FGS* female genital schistosomiasis, *HIV* Human immunodeficiency virus, *OR* odds ratio, *PCR* polymerase chain reaction, *VIA* Visual inspection with acetic acid

^a^21 with uninterpretable images; ^b^one urine vial arrived to LUMC empty; ^c^2 results missing from HPTN-271 (PopART) database

The prevalence of active *S. haematobium* infection was 6.3% (15/237) by urine microscopy, and 14.8% (35/237) by urine CAA (Table 1). *Schistosoma* PCR in any genital specimen (CVL, cervical or vaginal swab) was positive in 14/237 (5.9%). Relative proportions for each FGS diagnostic being positive compared to all others are represented in sup. Table 1. Colposcopy images were interpretable in 216 women, of whom 70 had ‘*visual FG*S’ (29.5%). HIV-1 prevalence was 23.8% (56/235).

**Fig. 2:**
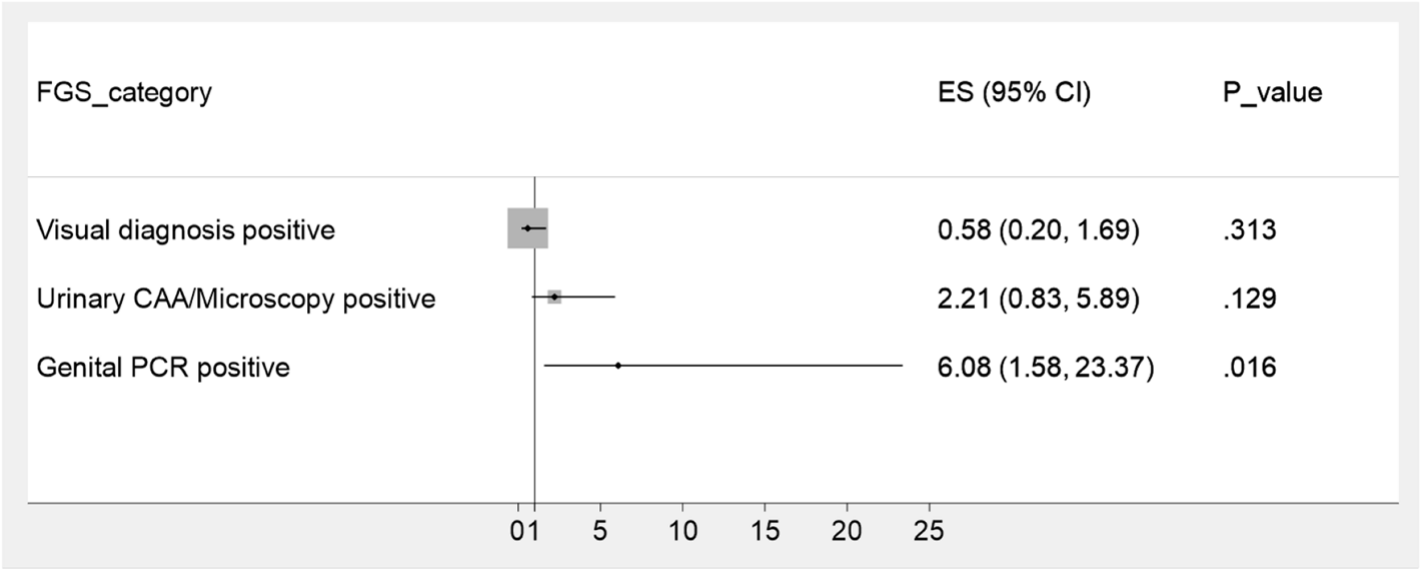
Forest Plot illustrating the results of a multivariable logistic regression of different FGS diagnostic methods and positive visual inspection with acetic acid (VIA), odds ratios adjusted for age and HIV status

Of 14 women with positive *Schistosoma* PCR, 4 were VIA positive (28.6%), compared to 20 of 223 with negative PCR (9.0%). The adjusted odds ratio for the association between positive VIA and FGS diagnosed by genital PCR was 6.08 (95% CI: 1.58–23.37) and there was strong evidence for this association (*P =* 0.016). There was no evidence for an association between VIA positivity and ‘*visual FG*S’ (OR: 0.58, 95% CI: 0.20–1.69, *P* = 0.30) or urine CAA/microscopy (OR 2.21, 95% CI 0.83–5.89, *P =* 0.13) (see Fig. 2). There was only one woman who was both HIV-1 positive and FGS PCR positive and therefore it was not possible to perform further analysis. Of the 13 FGS positive women, 3 were HIV-negative (23.0%), and of the 55 FGS negative women 7 were HIV positive (12.7%).

## Discussion

**Fig. 3:**
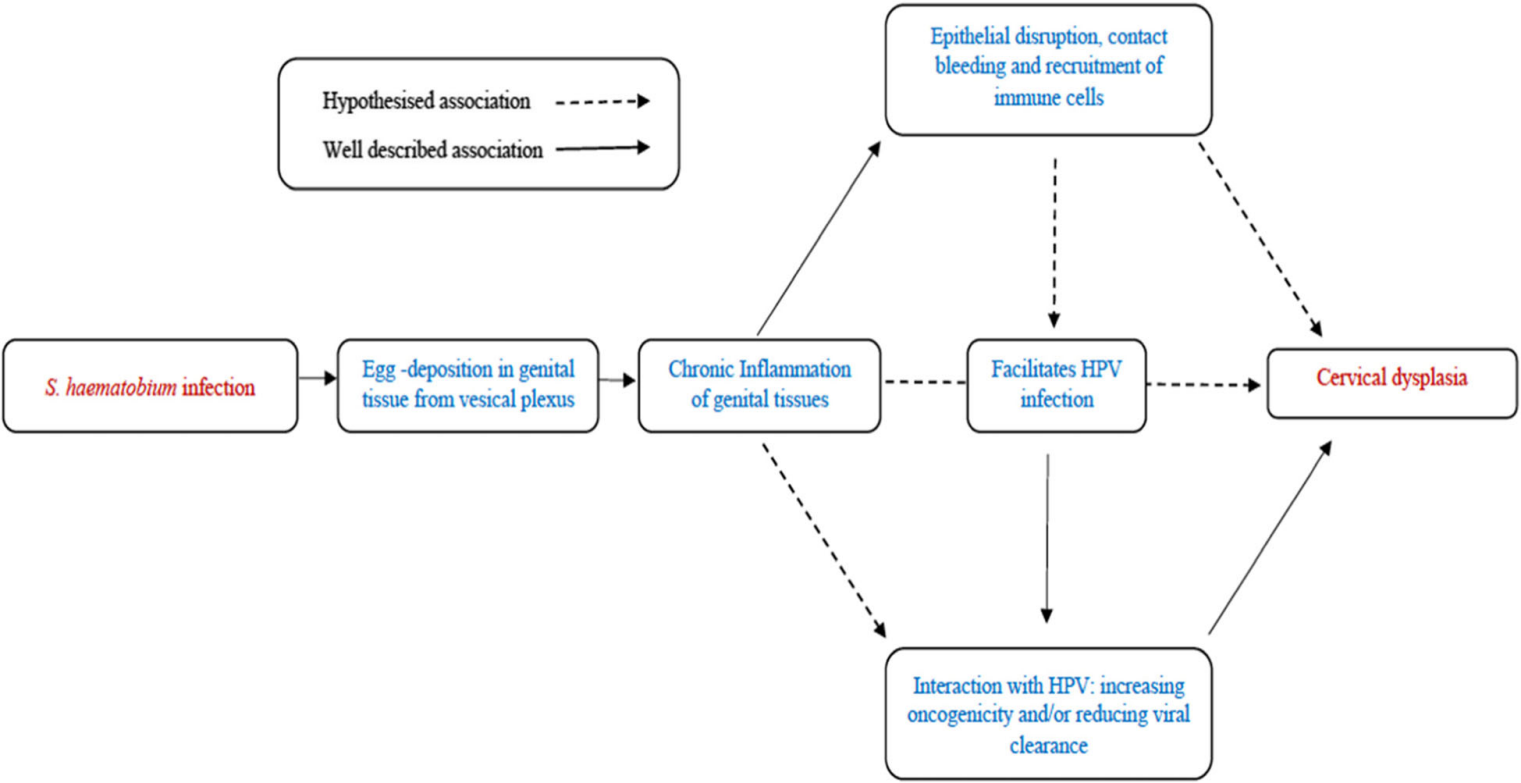
Conceptual pathway highlighting possible mechanisms linking female genital schistosomiasis and cervical dysplasia

This study is the first to provide evidence for an association between FGS and cervical dysplasia. These are two common gynaecological conditions in SSA, which together lead to significant morbidity and mortality. Chronic inflammation due to *S. haematobium* egg deposition is thought to be on the causal pathway to bladder squamous cell carcinoma [7]. We hypothesize that the inflammatory consequences of *S. haematobium* egg deposition in FGS could similarly contribute to cervical cancer pathogenesis (Fig. 3). Possible mechanistic synergies with co-infections include FGS-related epithelial disruption [32] allowing HPV to establish infection, and the recruitment of immune cells that may influence the interaction of HIV or HPV with cervical tissue. Additionally, changes in local pro-inflammatory cytokines may promote HPV persistence or transcription [33] and a systemic Th2 environment may be associated with cervical lesion progression [34]. Alternatively, there could be confounders at the community level including access to healthcare, health education and socio-economic factors. Of note, we did not find an association between VIA and previous self-reported history of schistosomiasis. While participants may have been asymptomatic or have limited access to diagnostics, we were unable to confirm a previous diagnosis of bilharzia, and so this variable is subject to recall bias. The possible association between FGS and cervical dysplasia has important clinical implications in endemic areas where FGS is not yet considered a risk factor for cervical cancer [35]. If this association is substantiated FGS diagnosis and treatment could become an important component of cervical cancer prevention.

Several previous studies have investigated the association between *S. haematobium* and cervical dysplasia. However, heterogeneous methods in diagnosing both the exposure (FGS) and the outcome (cervical dysplasia) make cross-study comparisons challenging. In a South African study, no association was found between FGS diagnosed by CVL PCR, or pap-smear and cervical dysplasia diagnosed with cytology. However, over 97% of pap-smears from women with FGS were uninterpretable, limiting the sample size [23]. A population-based cross-sectional study in Zimbabwe showed an association between the presence of typical FGS homogenous yellow sandy patches and high-risk HPV [36]. After 5 years of follow-up in this cohort, sandy patches were not associated with high risk HPV persistence or cervical dysplasia but again, conclusions were limited by low power [22].

To our knowledge, VIA has not previously been used for the diagnosis of cervical dysplasia in the context of FGS. A meta-analysis found a pooled sensitivity of 78% and specificity of 88% for CIN2+/high-grade squamous intraepithelial lesion detection by VIA as compared to colposcopy [37], and was preferable in terms of resources and availability [38]. In our cohort we found a low uptake of cervical cancer screening (44.9%), which has previously been demonstrated in Zambia [39]. This may reflect known barriers to cervical cancer screening such as limited health education and stigma [40]. Indeed, a 2016 cross-sectional study in Lusaka, Zambia found that only 36.8% of participants had heard of cervical cancer and only 20.7% of women had ever attended screening [41]. VIA has been extensively used in Zambia and has been shown to be effective in reducing cervical cancer related mortality [42], and can be effectively scaled up to reach a higher proportion of the population [43] .

This study included young women aged 18–31. There is a lack of evidence surrounding age of initiation for cervical screening, in part due to reduced cervical cancer incidence in women under 25 [4], lesion regression in younger women [44], and potential of harm with cervical interventions [45]. Zambian guidelines suggest screening initiation at 25 and WHO recommends screening HIV-positive women regardless of age [46]. Due to high HIV prevalence, all women presenting to Livingstone Central Hospital are offered VIA screening [27]. Including only younger women in this study may limit its generalisability and further investigation including older women may be required. Increased risk of cervical cancer in women with HIV-1 is well documented [47] however, this study was not powered to detect such an association. Our study did not collect data on previous cervical cancer screening, which is recommended for all HIV-1 positive women [48]. It is therefore possible that HIV positive women had previously been screened and treated.

Women in our study easily accessed the cervical cancer screening programme offered in the same clinic, illustrating how FGS diagnostics could be integrated into existing programmes. There is future potential for the diagnosis and treatment of two common and morbid gynaecological conditions at a single clinic visit. Given the cross-sectional study design, we cannot ascertain causality between FGS and cervical dysplasia. Lack of HPV testing, and absence of tissue biopsy to confirm VIA results also limit our conclusions. A longitudinal study is needed to investigate a temporal relationship between FGS, HPV, and cervical dysplasia across age groups and geographic locations. Mechanistic studies are also needed to elucidate the potential synergy between *S. haematobium* and HPV in cervical dysplasia. However, VIA remains a real-world diagnostic approach in low and middle income countries and is the recommended WHO screening modality in these settings [17].

In conclusion, this study shows an association between FGS, diagnosed by genital PCR, and VIA-positivity. Further research is needed to evaluate the association between FGS and cervical dysplasia and cervical cancer. If a causal link is established, FGS diagnosis and treatment may provide an additional opportunity to help reduce the burden of cervical cancer and achieve the WHO 2030 goal of cervical cancer elimination.

## Supplementary Information

**Additional file 1.**

## Data Availability

Due to the sensitive nature of the data collected in the BILHIV study, data will be available upon request by contacting the study PI Dr. Amaya Bustinduy. The data will be available on LSHTM Data Compass. Data will available on request, which is advised by the LSHTM information management team. The data will be available by request on LSHTM Data Compass.
