# Supplementary material for: Association between cervical dysplasia and female genital schistosomiasis diagnosed by genital PCR in Zambian women
Source: BMC Infect Dis. 2021 Jul 17;21:691. doi: 10.1186/s12879-021-06380-5 (PMC8286581; doi:10.1186/s12879-021-06380-5)
Supplement: Supplementary file 1 — Additional file 1. [file 12879_2021_6380_MOESM1_ESM.docx]

Supplementary table 1: Matrix to show the association between different diagnostic methods for urogenital schistosomiasis (CAA, urine microscopy) and FGS specifically (PCR and imaging)

|  | FGS by PCR | FGS by imaging | CAA positive | Urine microscopy positive |
| --- | --- | --- | --- | --- |
| FGS by PCR  N=14 |  | 5/14 (36%) | 7/14 (50%) | 9/14 (60%) |
| FGS by imaging  N=70 | 5/70 (7%) |  | 13/69 (19%) | 6/70 (9%) |
| CAA positive  N=35 | 7/35 (20%) | 13/35 (37%) |  | 11/35 (31%) |
| Urine microscopy  N=15 | 9/15 (60%) | 6/15 (40%) | 12/15 (80%) |  |
